# Complete Genome Sequence of Encapsulated *Haemophilus influenzae* Type f KR494, an Invasive Isolate That Caused Necrotizing Myositis

**DOI:** 10.1128/genomeA.00470-13

**Published:** 2013-10-03

**Authors:** Yu-Ching Su, Franziska Hörhold, Birendra Singh, Kristian Riesbeck

**Affiliations:** Medical Microbiology, Department of Laboratory Medicine Malmö, Lund University, Malmö, Swedena; Institute of Computer Science, Department of Mathematics and Computer Science, Friedrich-Schiller-University of Jena, Jena, Germanyb

## Abstract

*Haemophilus influenzae* serotype f (Hif) is an etiologic agent of bacterial invasive disease. Here, we report the first annotated genome sequence of the Hif strain KR494, which was isolated from a patient suffering from sepsis and necrotizing myositis. The genome sequence will increase the understanding of Hif pathogenesis.

## GENOME ANNOUNCEMENT

*Haemophilus influenzae* is a pathogen restricted to humans, and it resides in the upper respiratory tract, causing airway infections and, occasionally, invasive disease. Most systemic *Haemophilus* infections are associated with encapsulated strains, and *H. influenzae* serotype b (Hib) has been the most invasive serotype. However, since the introduction of a vaccine against Hib in the 1990s, serotype f (Hif) is now the most common invasive encapsulated *Haemophilus* species (1, 2). Hence, the clinical importance of Hif is emerging (3–6). The genomes of Hib strain 10810, *H. influenzae* serotype d (Hid) strains Rd Kw20 and Aw, and nontypeable *H. influenzae* (NTHi) strains were previously sequenced and annotated (7, 8). Here, we report the first fully annotated genome sequence of a Hif strain (KR494). Hif KR494 was recently isolated and caused necrotizing myositis in a previously healthy 70-year-old male (4). Hif KR494 possesses several virulence traits, as the isolate is serum resistant and adheres to and readily invades epithelial cells (4).

The draft genome sequence of Hif KR494 was obtained using Illumina 100-bp paired-end technology (HiSeq 2000; Illumina, CA). The total 8,888,894 reads (~400× coverage) were assembled using SOAP*denovo* 1.05 (9), resulting in 272 contigs that were further combined into 12 scaffolds (frame sequence). Closely related reference sequences (Hib 10810 [GenBank accession no. NC_016809] and Hid Rd Kw20 [GenBank accession no. NC_000907]) were used to complete the assembly through the alignment between the reference and frame sequences. PCR gap closure was performed for highly complex regions. Gap filling and single-base proofreading of the assembly results were performed by SOAPaligner/soap2 (9). Finally, a complete genome sequence map containing 1,856,176 bp was achieved. The *de novo* gene prediction was conducted with Glimmer 3.02 (10). Functional annotation was accomplished by BLAST analysis of the predicted genes with the Kyoto Encyclopedia of Genes and Genomes (11), Clusters of Orthologous Groups (12), Swiss-Prot, TrEMBL (13), NCBI (14), and Gene Ontology (15) databases. The rRNA, tRNA, and small RNA (sRNA) genes were predicted using RNAmmer (16), tRNAscan (17), and Rfam (18), respectively.

The Hif KR494 genome consists of only chromosomal DNA without any plasmids. The average G+C content of the genome is 38.05% (38.68% in the genes and 33.18% in the intergenic regions). The genome is composed of 1,742 putative protein-coding genes with an average length of 913 bp. The Hif genome contains 6 rRNA operons and 58 tRNAs. The majority of the annotated genes are involved in amino acid metabolism (9.6%) and protein translation (9.0%). Genes were found for most of the major known *Haemophilus*-related virulence factors, i.e., *Haemophilus* adhesion and penetration protein (Hap), opacity-associated protein A (OopA), IgA1 protease, *Haemophilus* surface fibril (Hsf), proteins D, E, and F, and genotype IIIb fimbriae (19–24). The gene locus *hmw1A1B*, encoding high-molecular-weight adhesion protein, and common *Haemophilus* antibiotic resistance genes (*bla*, *cat*, and *tet*) were absent. More detailed characterizations of the KR494 genome, including pangenomic analysis with other *H. influenzae* serotypes and the identification of Hif exclusive genomic features, are currently in progress.

### Nucleotide sequence accession number.

The complete genome sequence of *H. influenzae* KR494 has been deposited in GenBank under the accession no. CP005967.
